# The Role of the Gut Microbiota in Heart Failure: Pathophysiological Insights and Future Perspectives

**DOI:** 10.3390/medicina61040720

**Published:** 2025-04-14

**Authors:** Francisco Epelde

**Affiliations:** 1 Internal Medicine Department, Hospital Universitari Parc Taulí, 08208 Sabadell, Spain; fepelde@gmail.com; 2 Medicine Department, Facultad de Medicina, University Autonoma of Barcelona, 08193 Bellaterra, Spain

**Keywords:** gut microbiota, heart failure, gut–heart axis, dysbiosis

## Abstract

The gut microbiota has emerged as a crucial player in cardiovascular diseases, including heart failure (HF). Recent studies have highlighted the bidirectional interaction between the gut and the heart, often referred to as the gut–heart axis. Dysbiosis, characterized by alterations in microbial composition and function, has been linked to systemic inflammation, metabolic disturbances, and impaired cardiovascular homeostasis. This review explores the mechanisms through which gut microbiota influences HF, including microbial metabolite production, inflammatory pathways, endothelial dysfunction, hormonal modulation, fluid retention, and sodium absorption. The potential therapeutic implications of microbiota modulation through diet, probiotics, and pharmacological interventions are also discussed. Understanding these mechanisms could pave the way for novel diagnostic and therapeutic strategies in the management of HF. Future research should focus on longitudinal studies to establish causality and the development of personalized microbiota-based interventions.

## 1. Introduction

Heart failure (HF) remains a major global health challenge, with increasing prevalence and significant morbidity and mortality [1]. Despite advances in pharmacological and interventional therapies, HF continues to impose a substantial burden on healthcare systems [2]. Traditional risk factors, including hypertension, diabetes, and atherosclerosis, have been well established [3]. However, emerging evidence suggests that the gut microbiota may play a pivotal role in HF pathophysiology [4]. The gut microbiota, comprising trillions of microorganisms residing in the gastrointestinal tract, exerts systemic effects through metabolite production, immune modulation, hormonal regulation, and fluid balance [5]. Disruptions in microbiota composition, known as dysbiosis, have been implicated in cardiovascular diseases, including HF [6]. This review aims to elucidate the intricate relationship between the gut microbiota and HF, focusing on underlying mechanisms and potential therapeutic strategies [7].

### 1.1. Factors Influencing Gut Microbiota Composition

The composition of the gut microbiota is influenced by a variety of factors, including diet, age, genetics, medication use, and environmental exposures [8]. Dietary patterns play a crucial role in shaping microbial diversity and function [9]. Diets rich in fiber, polyphenols, and fermented foods promote the growth of beneficial bacteria that produce short-chain fatty acids, which support gut barrier integrity and reduce systemic inflammation [10]. Conversely, diets high in saturated fats, refined sugars, and processed foods have been associated with dysbiosis, favoring the proliferation of pro-inflammatory bacterial species that contribute to metabolic disturbances and cardiovascular dysfunction [11].

Age is another important determinant of microbiota composition [12]. Infants are born with a relatively sterile gut that becomes colonized by maternal and environmental microbes during early life [13]. As individuals age, microbiota diversity typically increases, reaching a stable state in adulthood [14]. However, aging is associated with shifts in microbial populations, often characterized by a decline in beneficial bacteria and an increase in pathogenic species [15]. These changes have been linked to heightened inflammation and an increased risk of chronic diseases, including HF [16].

Genetics also play a role in shaping microbiota composition, with studies indicating that host genetic factors influence the abundance of specific microbial taxa [17]. However, environmental factors and lifestyle choices appear to have a greater impact on microbiota variability [18]. Medication use is another critical factor, as antibiotics, proton pump inhibitors, and nonsteroidal anti-inflammatory drugs can alter microbial diversity and lead to persistent dysbiosis [19]. Cardiovascular medications, including beta-blockers and angiotensin-converting enzyme inhibitors, have also been shown to interact with the gut microbiota, potentially modifying their therapeutic effects [20].

Environmental exposures, including pollutants and stress, further contribute to microbiota composition [21]. The human microbiota, a diverse community of bacteria, fungi, and viruses residing primarily in the gut, plays an essential role in immune regulation, metabolism, and disease prevention. While diet and genetics have long been considered the primary determinants of microbiota composition, a growing body of research highlights the profound impact of environmental exposures on microbial diversity and function. From air pollution and pesticides to urbanization and climate change, environmental factors shape the microbiota in ways that can either promote health or contribute to disease susceptibility.

One of the most concerning environmental influences on the microbiota is air pollution, particularly fine particulate matter (PM2.5), nitrogen dioxide (NO_2_), and other airborne contaminants. Studies have demonstrated that chronic exposure to polluted air can alter the gut microbiota, reducing beneficial bacterial populations while promoting the growth of pro-inflammatory species. This dysbiosis has been linked to increased systemic inflammation, metabolic disorders, and cardiovascular diseases. Animal models further suggest that inhaled pollutants may directly affect the gut via the gut–lung axis, where inflammatory responses initiated in the lungs influence intestinal microbial composition.

Similarly, exposure to pesticides, herbicides, and heavy metals can have lasting effects on microbiota health. Glyphosate, a widely used herbicide, has been shown to disrupt gut microbial balance by inhibiting bacterial enzymes involved in amino acid synthesis. The consequences extend beyond the gastrointestinal tract, as pesticide-induced dysbiosis has been implicated in neurodevelopmental disorders, immune dysfunction, and metabolic diseases. Heavy metals such as lead, cadmium, and arsenic also perturb microbial composition, often reducing microbial diversity and increasing gut permeability, thereby facilitating the entry of toxins into systemic circulation.

The built environment, including urbanization and industrialization, has also emerged as a key modulator of microbiota diversity. Individuals residing in urban settings tend to have lower microbial diversity compared to those living in rural or agrarian communities. This difference is partially attributed to reduced exposure to soil-based and animal-associated microbes, which play a role in immune system development. Furthermore, excessive antibiotic usage, ultra-processed diets, and lack of interaction with natural ecosystems contribute to a gradual decline in beneficial microbial species, potentially increasing susceptibility to autoimmune diseases, allergies, and inflammatory disorders.

Climate change further exacerbates these challenges by altering the ecology of microbial environments. Rising global temperatures, increased humidity, and shifting ecosystems influence microbial populations, affecting food production, water safety, and the spread of antibiotic-resistant bacteria. The growing prevalence of vector-borne diseases, such as Lyme disease and malaria, is partially driven by climate-induced changes in microbial habitats. Additionally, extreme weather events and natural disasters disrupt microbial ecosystems, impacting human health in ways that are only beginning to be understood.

Recognizing the interplay between environmental exposures and microbiota opens new avenues for mitigating health risks. Strategies such as reducing air pollution, limiting pesticide exposure, preserving natural biodiversity, and promoting dietary interventions rich in fiber and probiotics may help counteract microbiota disruption. Moreover, advances in microbiome-based therapies, including targeted probiotics and microbial transplants, could provide innovative solutions to restore microbial balance in individuals affected by environmental dysbiosis.

**Figure 1:**
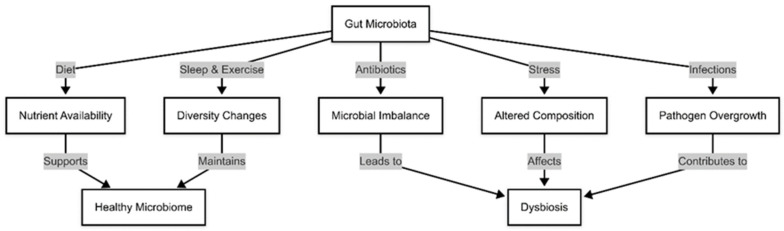
Factors that modify gut microbiota.

Chronic stress has been associated with alterations in gut permeability and microbial diversity, exacerbating systemic inflammation and neurohormonal dysregulation [22]. Understanding these factors is crucial for developing targeted interventions aimed at preserving microbiota diversity and preventing dysbiosis-related complications in HF patients [23]. These changes can be resumed in Figure 1.

### 1.2. Diseases Associated with Gut Microbiota Dysregulation

Gut microbiota dysregulation has been implicated in a variety of diseases beyond cardiovascular conditions, including metabolic disorders, gastrointestinal diseases, autoimmune conditions, and neurodegenerative disorders [24]. One of the most well-studied associations is with metabolic syndrome and type 2 diabetes [25]. Dysbiosis has been linked to insulin resistance through mechanisms involving increased gut permeability, chronic low-grade inflammation, and altered bile acid metabolism [26]. Microbial-derived metabolites, such as trimethylamine N-oxide and branched-chain amino acids, have been shown to impair glucose metabolism and contribute to the development of diabetes and obesity [27].

Inflammatory bowel diseases (IBD), including Crohn’s disease and ulcerative colitis, are strongly associated with gut microbiota imbalances [28]. Patients with IBD exhibit reduced microbial diversity, increased pathogenic bacteria, and altered immune responses [29]. Fecal microbiota transplantation and probiotics have shown promise in restoring microbial balance and alleviating symptoms, highlighting the potential of microbiota-targeted therapies in treating these conditions [30].

Autoimmune diseases, such as rheumatoid arthritis and multiple sclerosis, have also been linked to microbiota alterations [31]. The gut microbiota plays a crucial role in immune system regulation, and dysbiosis can lead to immune hyperactivation and systemic inflammation [32]. Studies suggest that microbial imbalances may contribute to the development of autoimmunity by altering antigen presentation and triggering inappropriate immune responses [33].

Neurodegenerative diseases, including Parkinson’s disease and Alzheimer’s disease, are increasingly recognized as having a gut–brain connection [34]. Dysbiosis in Parkinson’s disease has been associated with increased gut permeability, systemic inflammation, and accumulation of neurotoxic compounds [35]. In Alzheimer’s disease, microbial metabolites such as amyloid proteins may exacerbate neuroinflammation and cognitive decline [36]. Understanding these interactions may open new therapeutic avenues targeting the gut microbiota to prevent or slow neurodegenerative processes [37].

### 1.3. Gut Microbiota and Hydrosaline Retention

**Figure 2:**
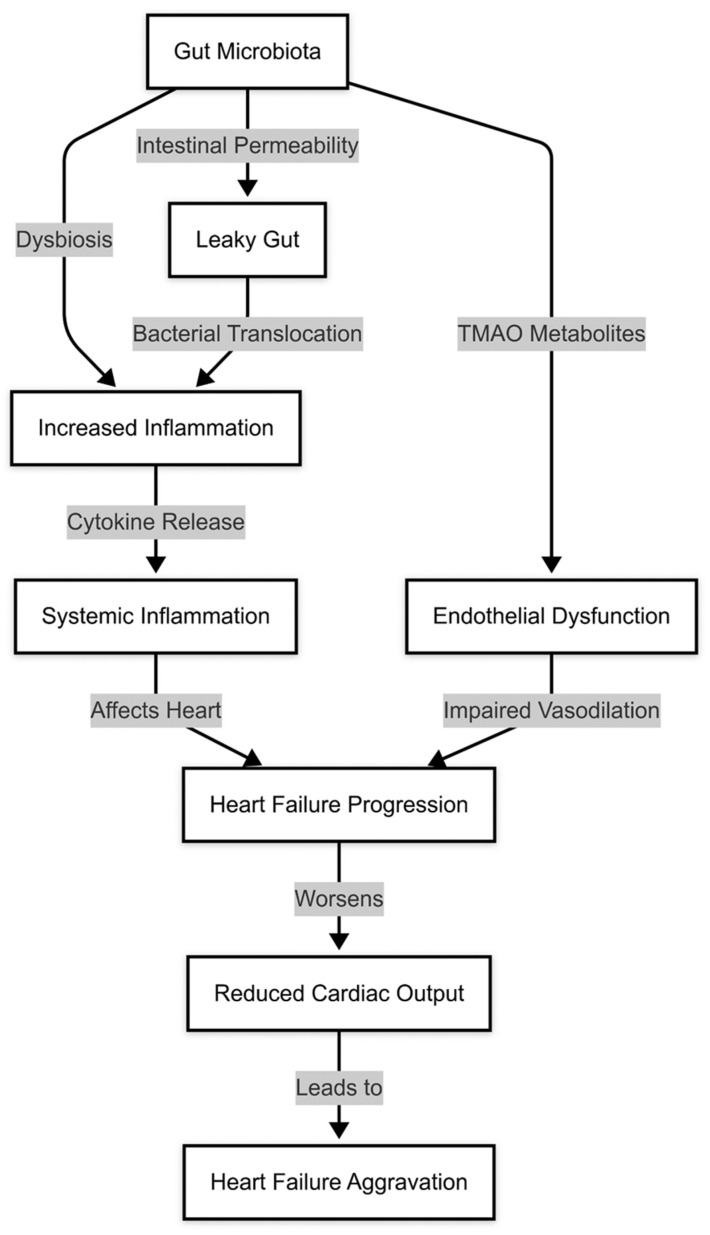
Gut microbiota dysbiosis and cytokine release.

The gut microbiota is increasingly recognized as a crucial factor in fluid and electrolyte homeostasis, particularly in the regulation of sodium and water retention, which are key contributors to heart failure progression [38]. Dysbiosis has been associated with an imbalance in microbial metabolites that influence renal sodium handling and fluid accumulation [39]. Certain bacterial species regulate the production of short-chain fatty acids, which have been shown to affect sodium transport mechanisms in the kidney [40]. When the microbiota is disrupted, this regulatory function can be impaired, leading to increased sodium reabsorption and subsequent water retention, exacerbating volume overload in HF patients [41]. These changes are resumed in Figure 2.

Furthermore, the interaction between gut microbiota and the renin–angiotensin–aldosterone system plays a significant role in hydrosaline retention [42]. Dysbiosis-induced inflammation leads to increased production of aldosterone, which promotes sodium retention in the kidneys, further contributing to fluid overload [43]. In addition, alterations in bile acid metabolism, another key function regulated by the gut microbiota, have been shown to impact sodium and water excretion [44]. Dysregulation of bile acid signaling can impair natriuresis, leading to increased sodium retention and worsening congestion in HF patients [45].

Clinical studies suggest that modifying the gut microbiota through probiotics, prebiotics, and dietary interventions may influence sodium and water balance, potentially alleviating congestion and improving HF symptoms [46]. However, more research is needed to determine the long-term impact of microbiota-targeted therapies on fluid retention and cardiovascular outcomes [47].

### 1.4. Gut Microbiota and Endothelial Dysfunction

The endothelium, a single layer of cells lining blood vessels, plays a pivotal role in vascular homeostasis. By producing nitric oxide (NO), it regulates vasodilation, prevents platelet aggregation, and protects against inflammatory injury. However, in endothelial dysfunction, NO bioavailability is reduced, leading to vascular stiffness, inflammation, and impaired blood flow regulation. While conventional risk factors such as hyperlipidemia and insulin resistance have long been implicated, a deeper examination reveals that microbial-derived metabolites can either protect or impair endothelial function.

One of the most studied microbial byproducts is trimethylamine-N-oxide (TMAO), a compound synthesized from dietary choline, lecithin, and carnitine, which are abundant in red meat, eggs, and dairy products. The gut microbiota converts these nutrients into trimethylamine (TMA), which is then oxidized in the liver to form TMAO. Elevated TMAO levels have been consistently linked to vascular inflammation, increased oxidative stress, and endothelial dysfunction. Experimental models have demonstrated that TMAO enhances foam cell formation, disrupts endothelial NO production, and increases arterial stiffness, leading to accelerated atherosclerosis. Clinical studies further corroborate these findings, showing that individuals with high circulating TMAO levels exhibit greater cardiovascular risk, independent of traditional risk factors.

In contrast to the deleterious effects of TMAO, another class of gut microbial metabolites—short-chain fatty acids (SCFAs)—serves as a protective counterpart. SCFAs, including butyrate, acetate, and propionate, are produced by the fermentation of dietary fibers by gut bacteria. These metabolites exert profound cardiovascular benefits by enhancing endothelial NO synthesis, reducing systemic inflammation, and maintaining vascular barrier integrity. In particular, butyrate has been shown to activate endothelial AMPK signaling, promoting anti-inflammatory and antioxidant effects that counteract endothelial dysfunction.

However, the composition of the gut microbiota determines the balance between harmful and beneficial metabolites. In the presence of gut dysbiosis, characterized by an overgrowth of pathogenic bacteria and depletion of SCFA-producing microbes, the endothelium becomes increasingly susceptible to dysfunction. One key mechanism linking dysbiosis to endothelial impairment is increased intestinal permeability, often referred to as “leaky gut”. When the gut barrier is compromised, lipopolysaccharides (LPS)—pro-inflammatory endotoxins from Gram-negative bacteria—enter systemic circulation. These endotoxins trigger endothelial inflammation, increase oxidative stress, and impair vascular relaxation, further exacerbating cardiovascular risk.

Emerging therapeutic approaches now focus on modulating the gut microbiota to restore endothelial function. Dietary interventions, particularly a Mediterranean or plant-based diet, have shown promise in shifting the microbial balance towards SCFA-producing bacteria while reducing TMAO synthesis. Additionally, targeted probiotics and prebiotics are being investigated for their ability to enhance NO bioavailability and reduce vascular inflammation. Specific probiotic strains, including Lactobacillus and Bifidobacterium, have demonstrated potential in attenuating endothelial dysfunction by modulating microbial metabolism.

Endothelial dysfunction is a key factor in the development of cardiovascular diseases, including heart failure, and the gut microbiota has been increasingly recognized as an important modulator of endothelial health [48]. The endothelium, which lines blood vessels, plays a critical role in regulating vascular tone, blood flow, and immune responses [49]. Dysbiosis can contribute to endothelial dysfunction through several mechanisms, including systemic inflammation, oxidative stress, and the production of harmful microbial metabolites [50].

One of the primary ways gut microbiota influences endothelial function is through the production of short-chain fatty acids (SCFAs), such as butyrate and acetate, which exert anti-inflammatory and vasodilatory effects [51]. When the gut microbial balance is disrupted, SCFA production is reduced, leading to increased vascular inflammation and impaired nitric oxide (NO) availability, a crucial mediator of endothelial function [52]. Additionally, dysbiosis has been associated with elevated levels of trimethylamine N-oxide (TMAO), a microbial metabolite derived from dietary choline and carnitine, which has been linked to increased oxidative stress, vascular stiffness, and endothelial dysfunction [53].

Lipopolysaccharides (LPS) from Gram-negative bacteria can also contribute to endothelial dysfunction by triggering systemic inflammation and activating toll-like receptor 4 (TLR4) signaling pathways, promoting endothelial cell damage and increasing vascular permeability [54]. The chronic inflammatory state induced by dysbiosis may further impair endothelial repair mechanisms, exacerbating atherosclerosis and increasing the risk of cardiovascular complications [55].

**Figure 3:**
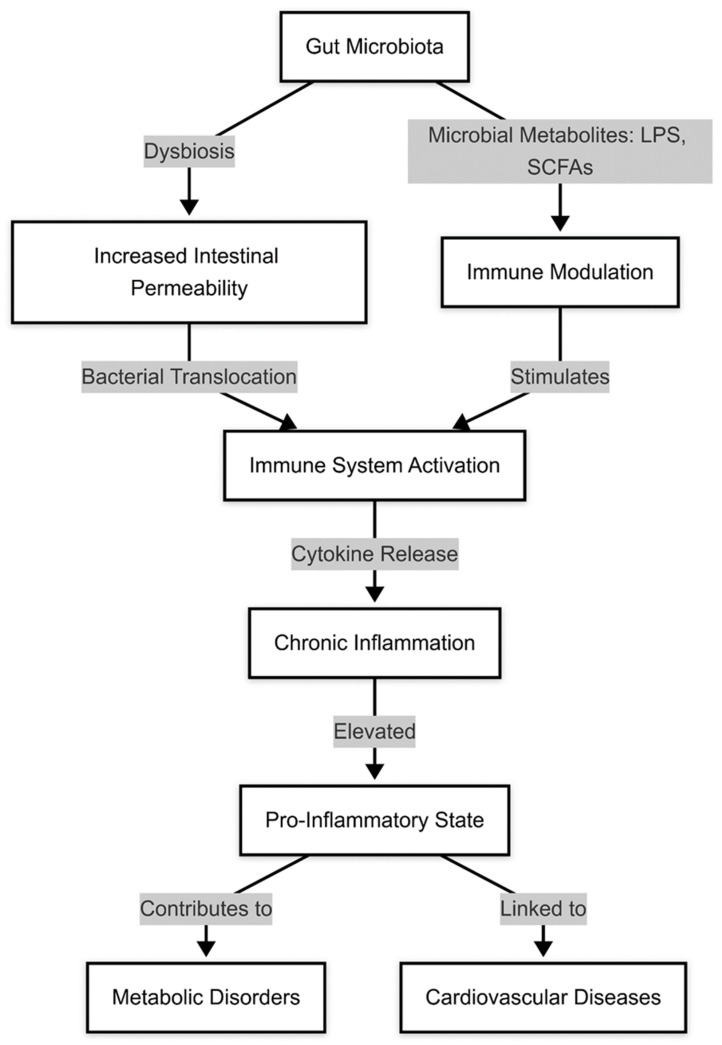
Gut microbiota, pro-inflammatory state, and heart failure.

Recent studies have suggested that interventions targeting the gut microbiota, such as dietary modifications, probiotics, and SCFA supplementation, may improve endothelial function and reduce cardiovascular risk [56]. However, more research is needed to establish the long-term benefits of microbiota-based therapies in endothelial health and HF progression [57]. A summary is provided in Figure 3.

The gut microbiota plays a crucial role in modulating systemic inflammation, a key factor in the pathogenesis and progression of heart failure [58]. Dysbiosis has been associated with increased levels of circulating inflammatory markers, including C-reactive protein, interleukin-6, and tumor necrosis factor-alpha, all of which contribute to endothelial dysfunction, myocardial fibrosis, and impaired cardiac function [59]. One of the primary drivers of this pro-inflammatory state is the increased translocation of lipopolysaccharides from G+ram-negative bacteria into the systemic circulation due to increased gut permeability, a phenomenon commonly referred to as endotoxemia [60]. This triggers a chronic inflammatory response that exacerbates cardiac remodeling and worsens HF outcomes [61].

In addition, gut microbiota-derived metabolites such as trimethylamine N-oxide (TMAO) have been shown to promote vascular inflammation, endothelial dysfunction, and thrombosis, further contributing to the deterioration of cardiovascular health [62]. Short-chain fatty acids, which normally exert anti-inflammatory effects, are often reduced in HF patients with dysbiosis, leading to an imbalance favoring pro-inflammatory pathways [63].

Recent studies suggest that microbiota-targeted therapies, including probiotic supplementation and dietary interventions aimed at increasing SCFA production, may help mitigate inflammation in HF patients [64]. While preliminary findings are promising, further research is needed to establish the long-term effects of microbiota modulation on inflammatory pathways and cardiovascular outcomes [65].

### 1.5. Gut Microbiota and Pharmacological Treatment in Heart Failure

The gut microbiota plays a significant role in the metabolism, absorption, and efficacy of cardiovascular medications used in heart failure treatment [66]. Alterations in gut microbial composition can influence drug bioavailability by modulating enzymatic activity and affecting intestinal permeability [67]. Among the most extensively studied cases of microbiota-mediated drug metabolism is digoxin, a cardiac glycoside frequently used in HF with reduced ejection fraction (HFrEF). Research has demonstrated that certain strains of Eggerthella lenta, an anaerobic gut bacterium, possess reductase enzymes capable of inactivating digoxin by converting it into its less bioactive dihydro form. This microbial transformation leads to inter-individual variability in drug response, with some patients exhibiting reduced therapeutic effects despite receiving standard dosages. Notably, the presence of E. lenta alone does not necessarily predict digoxin inactivation; rather, it is the expression of specific reductase genes, which are inducible under particular metabolic conditions. Strategies to mitigate this microbial effect, such as dietary modifications with arginine-rich foods, have shown promise in limiting E. lenta-mediated digoxin inactivation, thereby optimizing drug efficacy.

Beyond digoxin, angiotensin-converting enzyme (ACE) inhibitors, such as enalapril and ramipril, also undergo microbiota-mediated modulation. These prodrugs require enzymatic conversion to their active forms, a process influenced by microbial metabolism. Certain gut bacteria contribute to the hydrolysis and biotransformation of ACE inhibitors, potentially altering their pharmacokinetics and leading to variability in blood pressure control and afterload reduction. This microbial influence may help explain differential responses to ACE inhibitors observed among HF patients, despite standardized dosing regimens.

Similarly, beta-blockers, including metoprolol and carvedilol, are subject to microbiota-driven metabolic modifications. Traditionally metabolized via hepatic cytochrome P450 enzymes, beta-blockers are also affected by gut bacterial enzymatic activity, which influences their intestinal absorption, degradation, and systemic bioavailability. While some microbial populations enhance beta-blocker stability and uptake, others accelerate degradation, leading to reduced plasma concentrations and suboptimal therapeutic effects.

Statins, frequently prescribed for HF patients with concurrent dyslipidemia, also exhibit microbiota-mediated pharmacokinetic variability. Gut bacteria can alter statin metabolism through deconjugation reactions, reducing systemic drug levels and potentially compromising lipid-lowering efficacy. Given the pleiotropic effects of statins in cardiovascular disease, including anti-inflammatory and endothelial-protective properties, microbial interference with statin bioavailability may have broader implications for HF outcomes.

These findings underscore the growing importance of pharmacomicrobiomics, the study of microbiota-drug interactions, in cardiovascular medicine. Identifying patients with microbiota-driven drug metabolism alterations may enable precision pharmacotherapy, optimizing drug selection and dosing based on an individual’s microbial composition. Emerging interventions, including probiotics, prebiotics, and fecal microbiota transplantation (FMT), hold potential for modifying gut microbiota in a manner that enhances drug efficacy and reduces inter-patient variability [68].

One of the best-studied examples is digoxin, a cardiac glycoside used in HF, which can be inactivated by Eggerthella lenta, a bacterial species found in the gut [69]. Patients with a high abundance of this bacterium may experience reduced therapeutic efficacy of digoxin, highlighting the role of microbiota in interindividual variability in drug response [70]. Similarly, warfarin, a commonly prescribed anticoagulant, is influenced by microbial vitamin K production, which can affect its anticoagulant activity and necessitate dosage adjustments [71].

The gut microbiota also influences the metabolism of beta-blockers, which are widely used in HF management [72]. Some bacterial species have been shown to metabolize propranolol, reducing its bioavailability and potentially altering its therapeutic effects [73]. Moreover, dysbiosis-induced inflammation can affect cytochrome P450 enzyme activity in the liver, further impacting the metabolism of cardiovascular drugs [74].

Diuretics, another cornerstone of HF treatment, may also be affected by gut microbiota composition [75]. Dysbiosis-associated alterations in sodium and water absorption could influence diuretic efficacy, potentially leading to reduced natriuresis and fluid retention in HF patients [76]. Understanding these interactions is crucial for optimizing pharmacotherapy and minimizing variability in drug response [77].

Recent research has explored the potential of microbiota-targeted interventions, such as probiotics and prebiotics, to enhance drug metabolism and absorption [78]. Future studies should focus on characterizing specific microbial signatures that influence drug response and developing personalized therapeutic strategies that integrate gut microbiota modulation with HF pharmacological management [79].

Recent evidence suggests that patients with chronic heart failure, particularly those with reduced ejection fraction (HFrEF) and those suffering from HF with preserved ejection fraction (HFpEF) with high levels of systemic inflammation, may derive the greatest benefit from microbiota-targeted interventions. These individuals often exhibit gut dysbiosis, a condition characterized by a disruption in microbial diversity and an overgrowth of pathogenic bacteria. This imbalance contributes to the production of harmful metabolites such as trimethylamine-N-oxide (TMAO), a molecule derived from gut microbial metabolism of dietary choline and carnitine. Elevated TMAO levels have been strongly associated with increased atherosclerosis, endothelial dysfunction, and heightened cardiovascular mortality. Patients with high systemic inflammation, metabolic syndrome, and chronic kidney disease (CKD)—which frequently coexists with HF—may be particularly vulnerable to the detrimental effects of dysbiosis and are therefore prime candidates for microbiota-targeted therapies.

A range of microbiota-modulating strategies is currently under investigation. Among them, probiotics have demonstrated promising results in preclinical and early clinical studies. These live microorganisms, when administered in adequate amounts, can restore microbial diversity, reduce systemic inflammation, and improve gut barrier function. Certain strains of Lactobacillus and Bifidobacterium have been associated with lower TMAO production, reduced oxidative stress, and improved lipid metabolism. Although large-scale randomized clinical trials are still needed, preliminary evidence suggests that probiotic supplementation could complement conventional HF therapy, particularly in patients with heightened inflammatory markers.

Alongside probiotics, prebiotics, which serve as fermentable substrates for beneficial gut bacteria, have emerged as an adjunct strategy. Dietary fibers such as inulin and resistant starch promote the production of short-chain fatty acids (SCFAs), including butyrate, which exerts anti-inflammatory and cardioprotective effects. SCFAs enhance endothelial function, regulate blood pressure, and mitigate myocardial fibrosis, all of which are key contributors to HF progression. Given that HF patients frequently exhibit a diet low in fiber and high in processed foods, prebiotic supplementation represents a practical and non-invasive intervention to improve gut health and systemic inflammation.

Another compelling approach involves fecal microbiota transplantation (FMT), a procedure that transfers a healthy donor’s microbiota into the gastrointestinal tract of a recipient. While primarily used in the treatment of recurrent Clostridioides difficile infections, FMT has demonstrated potential in restoring microbial diversity in metabolic disorders. Though still in its early investigational phase for cardiovascular conditions, FMT could offer a radical means of reshaping the gut microbiota in HF patients with severe dysbiosis.

Dietary modifications also play a crucial role in microbiota modulation. A Mediterranean diet, rich in polyphenols, plant-based fibers, and omega-3 fatty acids, has been associated with increased microbial diversity and reduced cardiovascular risk. Given that dietary habits directly influence gut microbiota composition, integrating personalized nutritional counseling into HF management may enhance patient outcomes.

**Figure 4:**
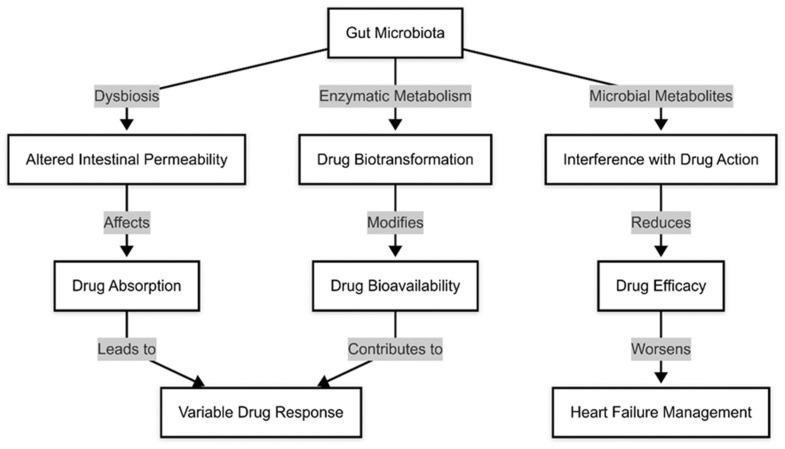
Gut microbiota and pharmacologic modifications.

Despite these promising interventions, challenges remain. The heterogeneity of gut microbiota across individuals necessitates personalized approaches tailored to each patient’s microbial profile. Moreover, long-term safety, optimal dosages, and potential interactions with pharmacological therapies must be further explored. Nevertheless, as our understanding of the gut–heart axis deepens, microbiota-targeted interventions may soon become a cornerstone of HF management, offering new hope for patients where conventional therapies fall short (Figure 4).

## 2. Conclusions and Future Perspectives

The gut microbiota is an increasingly recognized contributor to HF pathophysiology, influencing inflammation, endothelial function, neurohormonal regulation, atherosclerosis, drug metabolism, fluid retention, and sodium absorption [80]. Future research should focus on identifying specific microbial signatures associated with HF progression to develop more precise diagnostic biomarkers. Investigating the impact of microbiota-targeted therapies on long-term cardiovascular outcomes and integrating microbiome-based interventions into personalized medicine frameworks could revolutionize HF management. Large-scale clinical trials are needed to evaluate the safety and efficacy of prebiotics, probiotics, fecal microbiota transplantation, and novel pharmacological agents targeting gut-derived metabolites. Additionally, exploring the interactions between gut microbiota and commonly prescribed HF medications may provide insights into optimizing pharmacotherapy and reducing adverse effects. A multidisciplinary approach combining cardiology, microbiology, and precision medicine may pave the way for innovative strategies that harness gut microbiota modulation as a complementary tool in HF treatment, ultimately improving patient outcomes and reducing disease burden worldwide.
